# Impaired proteasomal degradation enhances autophagy via hypoxia signaling in Drosophila

**DOI:** 10.1186/1471-2121-14-29

**Published:** 2013-06-25

**Authors:** Péter Lőw, Ágnes Varga, Karolina Pircs, Péter Nagy, Zsuzsanna Szatmári, Miklós Sass, Gábor Juhász

**Affiliations:** 1 Department of Anatomy, Cell and Developmental Biology, Eötvös Loránd University, Pázmány P. s. 1/C, Budapest, H-1117, Hungary

**Keywords:** Autophagy, Drosophila, HIF-1α/sima, Hypoxia, p62/Ref2P, Proteasome

## Abstract

**Background:**

Two pathways are responsible for the majority of regulated protein catabolism in eukaryotic cells: the ubiquitin-proteasome system (UPS) and lysosomal self-degradation through autophagy. Both processes are necessary for cellular homeostasis by ensuring continuous turnover and quality control of most intracellular proteins. Recent studies established that both UPS and autophagy are capable of selectively eliminating ubiquitinated proteins and that autophagy may partially compensate for the lack of proteasomal degradation, but the molecular links between these pathways are poorly characterized.

**Results:**

Here we show that autophagy is enhanced by the silencing of genes encoding various proteasome subunits (α, β or regulatory) in larval fat body cells. Proteasome inactivation induces canonical autophagy, as it depends on core autophagy genes *Atg1*, *Vps34*, *Atg9*, *Atg4* and *Atg12*. Large-scale accumulation of aggregates containing p62 and ubiquitinated proteins is observed in proteasome RNAi cells. Importantly, overexpressed Atg8a reporters are captured into the cytoplasmic aggregates, but these do not represent autophagosomes. Loss of *p62* does not block autophagy upregulation upon proteasome impairment, suggesting that compensatory autophagy is not simply due to the buildup of excess cargo. One of the best characterized substrates of UPS is the α subunit of hypoxia-inducible transcription factor 1 (HIF-1α), which is continuously degraded by the proteasome during normoxic conditions. Hypoxia is a known trigger of autophagy in mammalian cells, and we show that genetic activation of hypoxia signaling also induces autophagy in Drosophila. Moreover, we find that proteasome inactivation-induced autophagy requires *sima*, the Drosophila ortholog of *HIF-1α*.

**Conclusions:**

We have characterized proteasome inactivation- and hypoxia signaling-induced autophagy in the commonly used larval Drosophila fat body model. Activation of both autophagy and hypoxia signaling was implicated in various cancers, and mutations affecting genes encoding UPS enzymes have recently been suggested to cause renal cancer. Our studies identify a novel genetic link that may play an important role in that context, as HIF-1α/sima may contribute to upregulation of autophagy by impaired proteasomal activity.

## Background

The proteasome is a multi-subunit protease complex, consisting of a 20S core particle and a 19S regulatory particle. 20S contains predominantly structural α and catalytic β subunits. 19S contains non-ATPase subunits that are involved in the recognition of polyubiquitinated proteins, and ATPase subunits responsible for unfolding substrates and maintaining protease activity. The ubiquitin-proteasome system (UPS) is considered to be the main route for the degradation of short-lived proteins. Proteasomal activity is also essential for eliminating excess or unfolded proteins and thus contributes to establishing steady-state overall protein levels. The selective and often well-timed degradation of regulatory proteins by the proteasome orchestrates and modulates multiple biological processes such as transcription, metabolism, cell cycle progression and differentiation [1].

During the main pathway of autophagy, initially appearing membrane cisterns called phagophores capture portions of the cytoplasm in double-membrane autophagosomes. These vesicles then deliver cargo for lysosomal degradation [2,3]. Autophagy is thought to be mainly responsible for degrading long-lived proteins and whole organelles, and stress-induced autophagy maintains the amino acid pool and energy production necessary for biosynthetic processes during chronic starvation [1,3].

UPS and autophagy were long considered as independent, parallel pathways. Recent observations challenged this view, suggesting interactions between these degradative routes [4,5]. First, autophagy also plays an important role in the degradation of ubiquitinated proteins. Loss of autophagy leads to neurodegeneration accompanied by the formation of pathological protein aggregates both in mice and flies [3,6,7]. These aggregates contain ubiquitinated proteins and p62 (also known as Ref2P in flies), an autophagy receptor. p62 facilitates selective autophagic removal of ubiquitinated cargo by binding to both ubiquitin and Atg8 that is covalently bound to phagophore and autophagosome membranes [8,9]. Second, protein degradation by the autophagy and UPS pathways is coordinately regulated by the evolutionarily conserved Foxo transcription factors in muscles [10,11]. In addition, we recently showed that genes encoding proteins of the UPS are upregulated in response to starvation in autophagy mutant Drosophila larvae compared to starved control animals [12]. Third, impairment of the UPS has been shown to enhance autophagy in various cells, but the molecular links are poorly characterized [4,5].

In this study, we investigated the consequences of proteasome inhibition on aggregate formation and autophagic activity in somatic clones of larval fat body cells in Drosophila. We showed that genetic activation of hypoxia signaling induces autophagy, and identified HIF-1α as a novel molecular link between UPS and autophagy.

## Results

### Depletion of genes encoding various proteasome subunits leads to impaired proteasomal degradation in vivo

**Figure 1:**
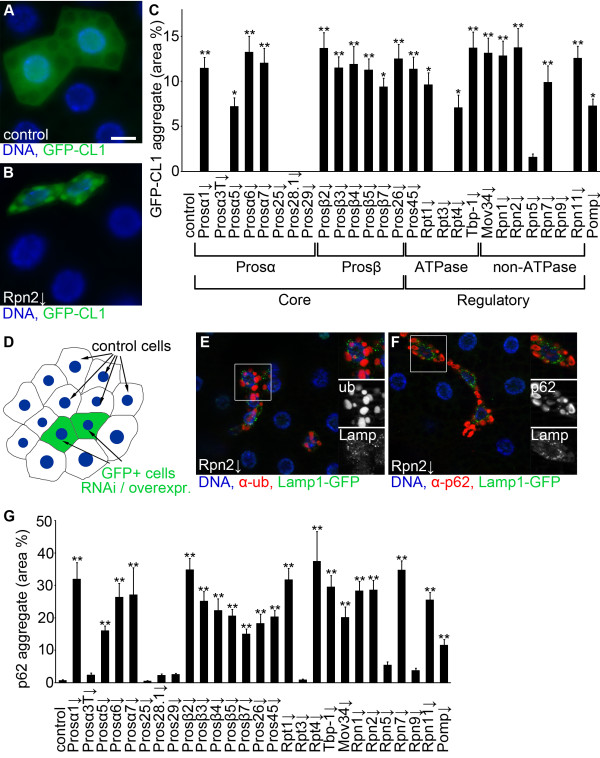
**Depletion of genes encoding various proteasome subunits decreases proteasomal activity in vivo. A)** Control cells expressing the proteasome degradation reporter GFP-CL1 are similarly sized as neighboring non-GFP control fat body cells and show diffuse fluorescence. Cell nuclei are labeled with DAPI (blue). **B)***Rpn2* RNAi cells are smaller than control cells and accumulate large aggregates of GFP-CL1. **C)** Proteasome RNAi leads to accumulation of GFP-CL1 aggregates relative to control cells shown in panel **A**. Statistically significant differences are marked by asterisks (Kruskal-Wallis test, n = 5-6 per genotype, ** P < 0.01, * P < 0.05), and error bars denote standard error. The different types of subunits are indicated as 20S core subunits (α and β), and 19S regulatory particle subunits (ATPase and non-ATPase). Pomp is required for assembly of the 20S core. **D)** Schematic of the clonal expression system. GFP-positive cells of interest are surrounded by wild-type cells in the same tissue of mosaic animals, serving as an internal control in various staining experiments. **E)** Depletion of *Rpn2* leads to accumulation of ubiquitinated protein aggregates in Lamp1-GFP marked fat body cell clones. Note that the numerous small Lamp1-GFP dots representing lysosomes are not ubiquitin-positive. **F)** Similarly, silencing of *Rpn2* leads to cell-autonomous accumulation of p62 aggregates in Lamp1-GFP marked fat body cell clones. Note that Lamp1-GFP dots are not p62-positive either. **G)** Proteasome RNAi leads to accumulation of endogenous p62 aggregates relative to control cells. n = 5-6 per genotype. Statistically significant differences are marked by asterisks (Kruskal-Wallis test, n = 5-6 per genotype, ** P < 0.01, * P < 0.05), and error bars denote standard error. Boxed areas in **E** and **F** are shown enlarged. Scale bar in **A** equals 20 μm for **A**, **B**, **E**, **F**.

We selected transgenic RNAi lines to mediate inducible silencing of 26 genes encoding different proteasome subunits in Drosophila. Expression of these UAS-proteasome RNAi constructs can be triggered in vivo in a tissue-specific manner by simply crossing these lines to another line that carries an appropriate promoter-Gal4 transgene (driver). As systemic RNAi mediated by an Actin-Gal4 driver resulted in developmental delays and lethality in case of the majority of RNAi lines, we used the standard mosaic approach to generate RNAi cells (marked by an appropriate fluorescent reporter such as GFP) in an otherwise wild-type background. To investigate proteasome activity in vivo, we analyzed transgenic flies expressing a fluorescent reporter of UPS function. GFP-CL1 is a fusion protein created by introducing a degradation signal to the otherwise stable green fluorescent protein (GFP) [13]. This protein is rapidly degraded by the UPS, and its steady state levels reflect the functional status of this pathway. In control animals, clones of fat body cells expressing GFP-CL1 were similar in size to neighboring cells and showed weak, diffuse GFP fluorescence (Figure 1A). Gal4-mediated expression of either one of 19 out of 26 different RNAi lines decreased cell size and led to the accumulation and large-scale aggregation of GFP-CL1, indicating impaired proteasomal degradation activity (Figure 1B, C, see also Additional file 1: Figure S1 for further images).

Proteasome maturation protein (Pomp) facilitates the main steps in 20S core complex formation at the ER by coordinating the assembly process, and it is necessary for the continuous supply of newly formed proteasomes. Similar to the above results, depletion of Pomp also resulted in accumulation of GFP-CL1 (Figure 1C).

We have also tested the level of p62, a well-characterized intracellular receptor for ubiquitinated proteins. As ubiquitin-containing proteins were shown to be captured into aggregates of p62 [9,14,15], inactivation of the proteasome was expected to increase p62 aggregation due to a block in ubiquitinated protein degradation. Note that in the various staining experiments in genetic mosaic animals, GFP-positive RNAi cells are always surrounded by control cells in the very same tissue, which serve as an internal control to directly compare phenotypes to (Figure 1D). Immunostainings indeed revealed large ubiquitin- and p62-positive aggregates in the cytoplasm of proteasome subunit RNAi cells compared to control non-GFP cells (Figure 1E, F, see also Additional file 2: Figure S2 for further images). Importantly, aggregates never colocalized with the lysosome marker Lamp1-GFP in these cells, suggesting that aggregated proteins are not accumulated inside lysosomes. Quantification of immunostainings revealed that 20 out of the 27 RNAi lines (targeting 26 different proteasome subunits and Pomp) led to large-scale accumulation of endogenous p62 aggregates (Figure 1G). Notably, exactly the same seven lines failed to trigger a statistically significant elevation of both p62 and GFP-CL1 aggregates. This may be due to that these RNAi lines did not work properly in fat body cells, although it cannot be excluded that some of the proteasome subunits targeted by these seven RNAi lines are dispensable for proteasome function. The transcription of p62 is also regulated during starvation and stress, both in Drosophila and mammalian cells [12,16,17]. RT-PCR experiments revealed that p62 transcription in proteasome depleted animals was also increased relative to controls (see Additional file 3: Figure S3).

It was obvious in these experiments that smaller fat body cells had higher GFP-CL1 levels (see Additional file 4: Figure S4). This suggested that proteasomal degradation is necessary for proper growth and endomitosis of larval fat body cells, and a more severe block of proteasome function likely produces smaller cells.

We selected five proteasome subunit RNAi lines for further experiments that all worked very well in both in vivo proteasome activity assays (that is, GFP-CL1 and p62 aggregation). These represent different subunit types: 20S core particle subunits Prosα1, Prosα5, Prosβ2, and 19S regulatory particle subunits Rpt1, Rpn2 (Figure 1C).

### Proteasome impairment enhances starvation-induced and basal autophagy

**Figure 2:**
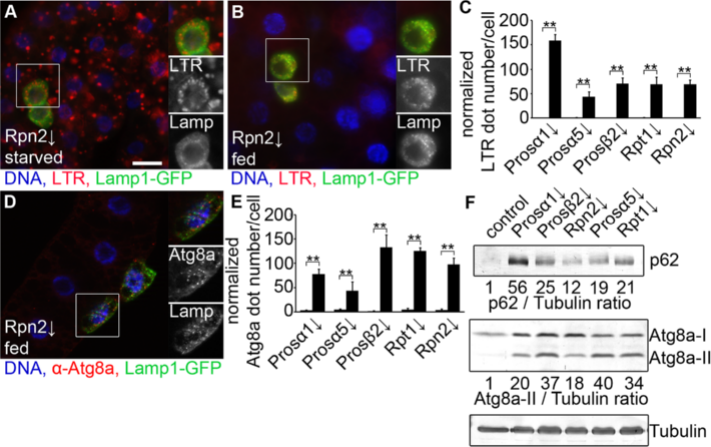
**Proteasome impairment enhances autophagy in *Drosophila *larvae. A)** Depletion of *Rpn2* increases punctate Lysotracker Red (LTR) staining in Lamp1-GFP marked larval fat body cell clones of starved animals relative to surrounding control cells. **B)***Rpn2* knockdown induces autophagy in fat body cell clones of well fed larvae, as LTR dots are only observed in GFP-positive RNAi cells but not in control cells. **C)** Silencing of genes encoding different proteasome subunits results in a statistically significant induction of punctate LTR in well fed conditions (u test, n = 5-7 per genotype, ** P < 0.01), and error bars denote standard error. **D)** Immunostaining reveals cell-autonomous activation of punctate endogenous Atg8a labeling in *Rpn2* RNAi cells, representing autophagosomes. **E)** Statistical evaluation of the effect of proteasome subunit RNAi on punctate endogenous Atg8a. Statistically significant differences are marked (u or *t* test, n = 5 per genotype, ** P < 0.01), and error bars denote standard error. **(F)** Western blots show that RNAi knockdown of genes encoding different proteasome subunits greatly increases the levels of both the specific autophagy cargo p62 and autophagosome-associated lipidated Atg8a-II. Numbers refer to relative expression levels compared to Tubulin, as determined by densitometric evaluation. Boxed areas in **A**, **B** and **D** are shown enlarged. Scale bar in **A** equals 20 μm for **A**, **B**, **D**.

Lysotracker Red (LTR) staining is an established and widely used assay for detection of autolysosomes in Drosophila fat body and other tissues [6,11,12,18-25]. Inactivation of proteasome function increased autophagic activity in Lamp1-GFP-marked fat body cell clones of starved larvae, based on increased punctate LTR labeling compared to surrounding non-GFP control cells (Figure 2A, see also Additional file 5: Figure S5 for further images). LTR structures also colocalized with Lamp1-GFP in clone cells, an artificial reporter that labels late endosomes and all lysosomes in Drosophila cells [26]. Note that the colocalization is not complete, as LTR only stains acidic, actively digesting lysosomes, whereas primary lysosomes are also labeled by Lamp1-GFP. Moreover, Lamp1-GFP is continuously degraded in active lysosomes, so its levels are often strongly decreased in LTR structures (see Additional file 6: Figure S6 for colocalization of these markers in control fat body cells dissected from starved animals, and for colocalization data in control and proteasome RNAi cells).

These genetic manipulations also activated autophagy in a cell-autonomous manner in well-fed larvae, based on increased LTR and autophagosome-associated punctate anti-Atg8a immunostainings (Figure 2B-E, see also Additional file 7: Figure S7 for further images). Similarly, knockdown of genes encoding proteasome subunits led to elevated levels of autophagosome-associated, lipidated Atg8a-II in western blots of well-fed larvae (Figure 2F). Levels of the ubiquitin-binding selective autophagy cargo p62 also showed a marked increase in these samples (Figure 2F).

We found that upon proteasome RNAi, smaller cells always had increased normalized LTR dot number (see also Additional file 8: Figure S8). Thus, more severe proteasome inactivation seems to induce higher levels of autophagy and a stronger cell growth impairment.

### Proteasome RNAi leads to accumulation of cytoplasmic aggregates and enhances autophagic flux

**Figure 3:**
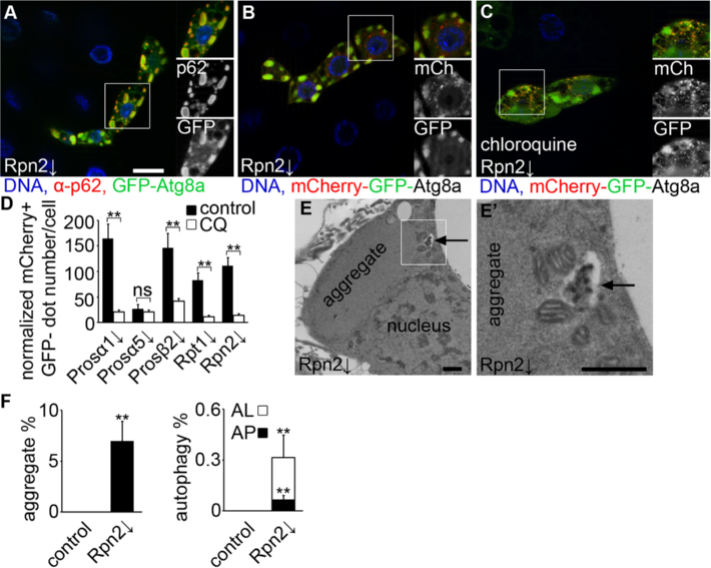
**Proteasome impairment leads to accumulation of cytoplasmic aggregates and enhances autophagic flux. A)** Overexpressed GFP-Atg8a reporter is incorporated into the large protein aggregates containing p62 in *Rpn2* RNAi cells. **B)** The tandemly tagged mCherry-GFP-Atg8a reporter forms numerous small mCherry-labeled autolysosomes, in addition to large aggregates positive for both mCherry and GFP in *Rpn2* RNAi cells. **C)** The lysosome inhibitor chloroquine blocks autophagy-dependent quenching of GFP, as now most puncta are positive for both mCherry and GFP. **D)** Quantification of data from panels **B** and **C** (u or t test, n = 5-8 per genotype, ** P < 0.01, * P < 0.05), and error bars denote standard error. **E)** Transmission electron microscopy reveals the presence of autolysosomes (arrow) and large protein aggregates in fat body cells with impaired proteasome function in well-fed larvae. The boxed area is shown enlarged in panel **E**’. **F)** Quantification of ultrastructural data. Protein aggregates occupy 7% of the total cytoplasm in *Rpn2* depleted fat body cells of well-fed larvae, and double-membrane autophagosomes (AP) and degrading autolysosomes (AL) take up 0.06% and 0.24% of the total cytoplasm, respectively. No such structures are recognized in fat body cells of well-fed control larvae (*u* test, n = 3 per genotype, ** P < 0.01), and error bars denote standard error. Boxed areas in **A**-**C** are shown enlarged. Scale bar in **A** equals 20 μm for **A**-**C**. Scale bars equal 1 μm in **E**, **E**’.

We have recently described that overexpressed p62 and Atg8a reporters bind each other to form large aggregates in Drosophila fat body cells [21]. Overexpressed GFP-tagged Atg8a, a routinely used marker of autophagosomes [6,18,23], not only labeled small structures likely representing genuine autophagosomes with a diameter of about 1 μm, but also colocalized with the large p62-positive aggregates (approximate diameter: 5 μm) that formed in proteasome RNAi cells (Figure 3A, see also Additional file 9: Figure S9 for further images). Importantly, small genuine autophagosomes marked by endogenous anti-Atg8a staining [20,25] showed markedly different size and distribution from this overexpressed Atg8a reporter in proteasome RNAi cells (compare Figure 2D with Figure 3A). We next employed a tandemly tagged mCherry-GFP-Atg8a reporter, a standard assay to follow autophagic flux [25,27]. This reporter is selectively transported to autolysosomes, where GFP is quenched while mCherry remains fluorescent. In addition to small mCherry-positive autolysosomes in the perinuclear region of proteasome RNAi cells, large structures positive for both GFP and mCherry were also formed, with a size and localization again reminiscent of protein aggregates (Figure 3B). Treatment with the lysosome inhibitor chloroquine prevented the quenching of GFP in acidic autolysosomes in proteasome RNAi cells, greatly decreasing the number of structures that were positive for only mCherry (Figure 3C and D, see also Additional file 10: Figure S10 for further images).

We then carried out electron microscopy to completely rule out the possibility that these large aggregates were inside autophagosomes. Ultrastructural images of proteasome depleted fat bodies dissected from well-fed animals revealed the presence of large cytoplasmic protein aggregates that were never surrounded by membranes (Figure 3E, F). In addition, double-membrane autophagosomes and degrading autolysosomes were also readily detected in well-fed larval samples, consistent with autophagy induction in these cells (Figure 3E, F). See also Additional file 11: Figure S11 for further ultrastructural images.

It was striking that upon proteasome RNAi, smaller cells always had increased p62 accumulation (see Additional file 12: Figure S12). These results altogether indicated that even though autophagic activity is enhanced in these cells, it fails to remove the excess protein aggregates formed due to impaired proteasomal degradation.

As expected, LTR practically never colocalized with Atg8a or p62 reporters (see Additional file 13: Figure S13). Even the size of these structures was different: LTR-positive digesting lysosomes in fat body cells had an approximate diameter of 1-3 μm, while p62 was present in the large protein aggregates (approximate diameter: 5 μm), and GFP-Atg8a labeled small autophagosomes (approximate diameter: 1 μm) in addition to being captured into the large p62 aggregates.

These data altogether suggest that p62 accumulation is due to both transcriptional and post-transcriptional mechanisms, that is, increased rate of transcription, and reduced protein turnover by autophagy due to large-scale aggregate formation.

### Activation of hypoxia signaling induces autophagy in Drosophila

**Figure 4:**
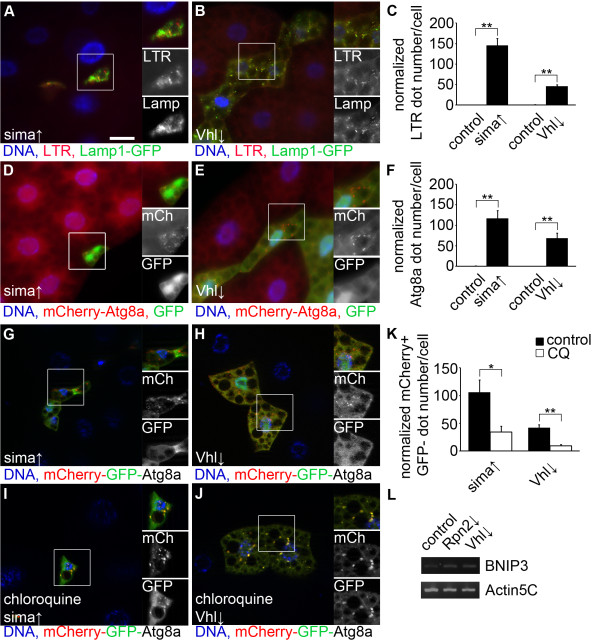
**Activation of hypoxia signaling induces autophagy in *Drosophila*. A**-**C)** Overexpression of the *Drosophila* HIF-1α ortholog sima **(A)** or depletion of *Vhl***(B)** induces the formation of autolysosomes positive for both Lamp1-GFP and LTR in fat body cell clones in well-fed larvae. Quantification of data is shown in panel **C**. Statistically significant differences are marked (*u* test, n = 6 per genotype, ** P < 0.01), and error bars denote standard error. **D**-**F)** Overexpression of sima **(D)** or knockdown of *Vhl***(E)** promotes the formation of punctate mCherry-Atg8a in fat body cell clones marked by GFP-nls (nuclear localization sequence) in well-fed larvae. Quantification of data is shown in panel **F**. Statistically significant differences are marked (u test, n = 6 per genotype, ** P < 0.01), and error bars denote standard error. **G**-**J)** Overexpression of sima **(G)** or depletion of *Vhl***(H)** leads to formation of dots that are mostly positive for mCherry with the mCherry-GFP-Atg8a reporter. Chloroquine treatment blocks the autolysosomal quenching of GFP, as puncta are now positive for both mCherry and GFP in cells overexpressing sima **(I)** or *Vhl* RNAi **(J)**. **K)** Quantification of data from panels **G-J**. Statistically significant differences are marked (u or *t* test, n = 5-8 per genotype, ** P < 0.01, * P < 0.05), and error bars denote standard error. **L)** RT-PCR analyses show that the transcription of BNIP3 is upregulated in both *Vhl* and *Rpn2* RNAi samples relative to controls. Boxed areas in **A**, **B**, **D**, **E**, **G**-**J** are shown enlarged. Scale bar in **A** equals 20 μm for **A**, **B**, **D**, **E**, **G**-**J**.

One of the best characterized substrates of UPS is the α subunit of hypoxia-inducible transcription factor HIF-1 [28]. HIF-1α is ubiquitinated by the tumor suppressor protein von Hippel-Lindau (VHL) under normoxic conditions, resulting in its proteasomal degradation. Decreased levels of oxygen lead to stabilization of HIF-1α by preventing its ubiquitination, and a concomitant upregulation of genes involved in the hypoxia response [28]. Hypoxia is a known trigger of autophagy in mammalian cells, but its role has not been characterized in Drosophila autophagy. The fly ortholog of the mammalian *HIF-1α* gene is *sima* (short for *similar*) [29]. Overexpression of sima, or depletion of *Vhl* led to the formation of LTR-positive autolysosomes in fat body cell clones of well-fed larvae (Figure 4A-C). Similarly, forced expression of sima or *Vhl* RNAi induced punctate mCherry-Atg8a representing autophagosomes and autolysosomes (Figure 4D-F). The mCherry-GFP-Atg8a reporter revealed that most of the punctate autophagic structures were positive for only mCherry in sima overexpressing and *Vhl* RNAi cells (Figure 4G, H). This indicated that GFP-Atg8a was delivered to autolysosomes and quenched. Chloroquine treatment again reduced the number of structures that were positive for only mCherry (Figure 4I-K), indicating that autophagic flux is enhanced in these cells.

We also carried out anti-active caspase 3 stainings to analyze cell death, but no apoptotic caspase activation was detected in cell clones undergoing proteasome or *Vhl* RNAi, or overexpressing sima in larval tissues (see Additional file 14: Figure S14)

### Proteasome inactivation-induced autophagy requires Atg genes and sima/HIF-1α

Since genetic activation of hypoxia signaling increased autophagy, we first tested whether proteasome RNAi activates this pathway. BNIP3 (BCL2/adenovirus E1B 19 kDa protein-interacting protein 3) is a well-characterized transcriptional target of HIF1 in mammalian cells [30]. Our RT-PCR experiments revealed that transcription of Drosophila *BNIP3* (*CG5059*) is enhanced upon silencing of *Rpn2* to a similar extent as in *Vhl* RNAi animals (Figure 4L). Additionally, proteasome RNAi increased the expression level of an in vivo transcriptional reporter for hypoxia signaling (see Additional file 15: Figure S15).

These results suggested that hypoxia signaling may be involved in proteasome RNAi-induced autophagy. To evaluate the genetic pathways mediating compensatory autophagy upon proteasome impairment, we tested the potential role of *sima*, core *Atg* genes required for starvation-induced autophagy, and *p62*.

**Figure 5:**
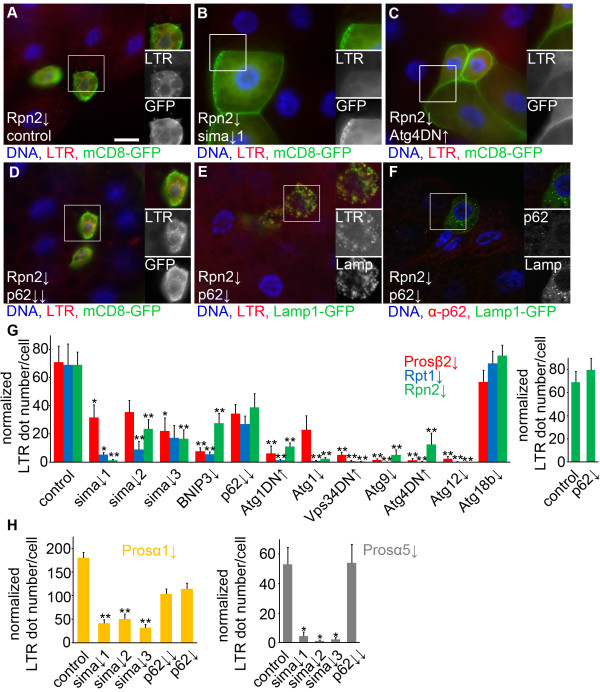
**Proteasome inactivation-induced autophagy requires *Atg *genes and *sima/HIF-1α*. A)** Depletion of *Rpn2* increases punctate LTR staining in larval fat body cell clones in fed animals, marked by membrane-associated mCD8-GFP. **B)** Simultaneous silencing of *Rpn2* and *sima* results in a block of punctate LTR staining. **C)** Overexpression of dominant-negative (DN) Atg4 inhibits LTR dot formation in *Rpn2* RNAi cells. **D**, **E)** Simultaneous silencing of *Rpn2* and *p62* using two independent RNAi lines combined **(D)** or a third independent single *p62* RNAi line **(E)** does not inhibit punctate LTR staining in larval fat body cell clones in fed animals. **F)** Depletion of *p62* blocks aggregate formation in *Rpn2* RNAi cells. **G)** Statistical evaluation of punctate staining in *Rpn2, Rpt1* and *Prosβ2* knockdown cells. Depletion of *sima* using 3 independent RNAi transgenes, silencing of *BNIP3, Atg1, Atg9, Atg12*, or overexpression of dominant-negative Atg1, Vps34, Atg4 inhibits proteasome inhibition-induced autophagy. Depletion of *p62* or *Atg18b* (which is dispensable for starvation-induced autophagy and used as a negative control here) does not block the autophagy-inducing effect of proteasome inactivation. Statistically significant differences are marked (Kruskal-Wallis test or ANOVA, n = 5-8 per genotype, * P < 0.05, ** P < 0.01), and error bars denote standard error. **H)** Similarly, silencing of *sima* but not *p62* attenuates LTR puncta formation in *Prosα1* and *Prosα5* RNAi cells. Statistically significant differences are marked (Kruskal-Wallis test or ANOVA, n = 5-8 per genotype, * P < 0.05, ** P < 0.01), and error bars denote standard error. Boxed areas in **A**-**F** are shown enlarged. Scale bar in **A** equals 20 μm for **A**-**F**.

Clones of fat body cells showed LTR-positive autolysosome formation in well-fed animals upon silencing of *Prosβ2*, *Rpt1* and *Rpn2* (Figure 5A, G). We silenced *sima* using 3 independent transgenic constructs, each of which targeted different regions of this gene and achieved knockdown by distinct experimental approaches (long hairpin- versus microRNA-based, please see Methods). Depletion of *sima* significantly inhibited proteasome inactivation-induced punctate LTR staining in most combinations (Figure 5B, G). Depletion of *Atg1, Atg9* or *Atg12* also blocked compensatory autophagy, similar to expression of dominant-negative Atg1, Vps34 or Atg4 transgenes (Figure 5C, G). As a negative control we used an RNAi line for *Atg18b*, as this gene was shown to be dispensable for starvation-induced autophagy in Drosophila (Figure 5G) [23]. Finally, we tested the role of *p62* using previously characterized transgenic RNAi lines [21]. Depletion of *p62* by combining two indepedent RNAi lines targeting different regions of the gene, or using a third transgenic RNAi line failed to suppress proteasome inactivation-induced punctate LTR staining: the changes were not statistically significant (Figure 5D, E, G). As expected, depletion of *p62* completely prevented the formation of p62 aggregates in proteasome inactivated cells (Figure 5F).

As a final confirmation of our results, we also tested two additional proteasome RNAi lines for hypoxia and p62 signaling. Depletion of *sima*, but not *p62*, also reduced the number of LTR puncta in *Prosα1* and *Prosα5* RNAi cells (Figure 5H).

## Discussion

Autophagy and UPS coordinately sustain cellular homeostasis through protein quality control and clearance. A wide range of neurodegenerative diseases (often described as proteinopathies) are characterized by accumulation of aggregate-prone proteins that are not efficiently removed by proteasome. Compensatory autophagy may alleviate disease pathology in these cases, as numerous studies showed that substrates of autophagy and UPS overlap [1,4,5]. Indeed, genetic or pharmacological activation of autophagy facilitates aggregate clearance and neuronal function in disease models [1,5]. Chronic block of proteasomal degradation also leads to toxicity, ER stress and cell death in various mammalian and Drosophila cells, and proteasome inhibitors are in clinical use to trigger apoptosis of cancer cells in multiple myeloma patients [31-34]. While we found no evidence of cell death in larval Drosophila fat body upon silencing of genes encoding essential subunits of the proteasome or genetic activation of hypoxia signaling, these genetic manipulations strongly enhanced both basal and starvation-induced autophagy in these cells, and this effect was dependent on canonical autophagy genes *Atg1, Vps34, Atg9, Atg4* and *Atg12*.

An important aspect of our study is that overexpressed tagged Atg8 is not ideal for analyzing autophagy in proteasome RNAi cells, as excess p62-containing aggregates incorporate these reporter molecules, likely due to the physical interaction of p62 and Atg8. This artefact may then lead to false conclusions regarding autophagic activity, similar to the case of co-overexpressed p62 and Atg8 reporters [21].

How does proteasome impairment lead to compensatory autophagy? Multiple scenarios are possible, and these are not mutually exclusive. One hypothesis would be that accumulation of excess cargo triggers the formation of autophagosomes. However, overexpression of specific autophagy substrates p62 or blue cheese (the fly homolog of human Alfy that is also involved in the clearance of ubiquitinated proteins) did not result in an obvious increase in autophagy in Drosophila, unlike proteasome impairment [21,35]. Moreover, p62 was found to be dispensable for both viability and starvation-induced autophagy in knockout mice [14]. Multiple selective autophagy receptors have been identified in mammalian cells, but these are relatively uncharacterized in Drosophila, as only HDAC6 and blue cheese have been analyzed so far besides p62 [13,35,36]. Further studies are necessary to find out whether any of the selective adaptors is involved in autophagy induced by proteasome inactivation, or by starvation. After all, autophagy is thought to be responsible for breakdown and recycling of bulk cytoplasm upon nutrient limitation [3], so it is possible that none of the receptors are actually required for these large-scale autolysosomal degradation processes.

A second hypothesis is that proteasomal degradation is essential to maintain free intracellular amino acid levels and compensatory autophagy is induced by amino acid shortage, as suggested in a recent publication [32]. In this paper, addition of exogenous amino acids was reported to attenuate proteasome inactivation-induced autophagy and partially rescue lifespan reduction in flies treated with a proteasome inhibitor [32]. In this scenario, loss of autophagy in cells with impaired proteasome activity would further reduce amino acid levels as both recycling pathways are inhibited, presumably resulting in a stronger block of cell growth. However, blocking the function of core autophagy genes did not further decrease the growth of proteasome RNAi cells in fat body clones, indicating that reduced amino acid levels are likely not the only link between these processes in this setting.

A third hypothesis is that stabilization of certain proteasome substrate(s) positively regulates autophagy. The levels of HIF-1α are normally kept low through ubiquitination mediated by VHL, which is followed by proteasomal degradation. Hypoxia leads to stabilization of HIF-1α, resulting in its translocation to the nucleus and upregulation of hypoxia-inducible genes that mediate survival at low oxygen concentrations [28]. Cytoprotective autophagy is also induced by hypoxia, and hypoxia-induced autophagy is necessary for survival of cancer cells in the central, poorly vascularized regions of solid tumors [30]. We found that genetic activation of hypoxia signaling induced autophagy in Drosophila, and silencing of the fly *HIF-1α* ortholog *sima* attenuated proteasome impairment-induced autophagy. These results suggest that autophagy is enhanced by proteasome inhibition at least in part through hypoxia signaling. A recent genome-wide association study identified mutations affecting the UPS pathway in renal cell carcinoma, which were associated with increased levels of HIF-1α in these tumors [37]. Interestingly, cells derived from many established cancers show elevated levels of autophagy, and multiple oncogenes and tumor suppressors (such as *VHL*) encode enzymes of the UPS involved in ubiquitin conjugation or deconjugation [38]. Thus, based on these and our results, activation of hypoxia signaling by UPS impairment may be responsible for increased autophagy in the absence of hypoxic conditions in a subset of established cancers.

## Conclusions

Our work showed that genetic inactivation of proteasomal degradation induces compensatory autophagy in the widely used Drosophila fat body model, which requires core autophagy genes *Atg1, Vps34, Atg9, Atg4* and *Atg12.* Proteasome impairment leads to large-scale accumulation of aggregates containing ubiquitinated proteins and the selective autophagy cargo p62, but *p62* itself turned out to be largely dispensable for enhanced autophagy. Finally, we showed that genetic activation of hypoxia/HIF-1α signaling induces autophagy in Drosophila, and HIF-1α/sima, a well-characterized proteasome substrate, contributes to autophagy induced by proteasome impairment.

## Methods

### Drosophila stocks and culture

Flies were raised at 25°C on standard cornmeal/agar media, at 50% humidity and a 12-hour light/12-hour dark daily cycle. Fly stocks used in this study were obtained from the Vienna Drosophila RNAi Center, VDRC, Vienna, Austria and Bloomington Drosophila Stock Center, Bloomington, Indiana, USA, or kindly provided by Udai Pandey (*GFP-CL1*) [13] and Pablo Wappner (*LDH-GFP*) [29] (see Additional file 16: Figure S16 for a list of stocks). Note that KK, GD and JF lines are long hairpin-based RNAi transgenics, and HMS stocks contain short microRNA-based silencing constructs.

### RT-PCR, western blots and immunostainings

Total RNA was isolated from L3 stage larvae using Purelink RNA Mini Kit (Ambion), followed by cDNA synthesis with Revertaid First Strand cDNA Synthesis Kit (Fermentas) and PCR amplification at 25 cycles. The following primers were used (designed to overlap exon-exon boundaries to prevent accidental amplification from genomic DNA): TACTCCTCCCGACACAAAGC, CTGGGTCATCTTCTCACGGT (Actin5C), TGGATCGACGCTGATAAAGA, GTCTCCTGAAACGGGCAAT (p62), CTTGGATCGAACTCAGCACA, CAATGTTCCAGCTACTGGGTG (BNIP3).

Western blots were carried out as described [6,25], using primary antibodies: rabbit anti-p62 (1:5,000) [21], rabbit anti-Atg8a (1:5,000) [25] or mouse monoclonal anti-Tubulin AA4.3 (1:1,000, DSHB), followed by alkaline phosphatase conjugated goat anti-rabbit, anti-mouse secondary antibodies (1:5,000, Millipore). Immunostainings were done as before [21,25], using primary antibodies chicken anti-GFP (1:1,500, Invitrogen), rabbit anti-Atg8a (1:500, gift of Katja Köhler) [20], rabbit anti-p62 (1:2,000) [21], rabbit anti-ubiquitin (1:50, Dako), rabbit anti-active caspase 3 (1:500, Cell Signaling). Secondary antibodies were Alexa 488 anti-chicken, Alexa 546 anti-rabbit (1:1,500, Invitrogen).

Please see Additional file 17: Figure S17 for original images of RT-PCR gels and western blots.

### Genetics and treatments

Clonal analysis in larval fat bodies was done as exhaustively described previously [6,18,19,21-25]. Briefly, cell clones were spontaneously generated by Flp-mediated excision of the FRT (Flp Recombination Target, >) cassette from Act > CD2 > Gal4 to activate UAS-dependent transcription of overexpression, dominant-negative and RNAi transgenes, together with the GFP reporters indicated in figure legends. Note that cell clones arose in embryos, and all of our analyses were carried out in L3 stage larvae at 84-90 hours after egg deposition. We also generated stable lines of *hsFlp[22], Act > CD2 > Gal4, UAS-mCD8-GFP* (all recombined together on chromosome X); *UAS-Rpn2* or *UAS-Rpt1* or *UAS-Prosβ2 RNAi* (located on autosomes). These lines were crossed to various *Atg*, *p62* and *sima* RNAi and dominant-negative lines in genetic interaction tests. RNAi was mediated in whole fat bodies by *cg-Gal4* for electron microscopy. As systemic knockdown of proteasomal genes resulted in early stage lethality, *hsFlp[22], Act > CD2 > Gal4, UAS-RNAi* stocks were heat shocked for 1 hour at 37 degrees at 48 hours after egg deposition to induce gene silencing in polyploid cells for western blots. Larvae of the age 60-66 hours after egg deposition were transferred to fresh medium containing 3 mg/ml chloroquine (Sigma) for a 24-hour treatment in autophagic flux experiments.

### Histology and imaging

Lysotracker stainings were carried out as described [19,21-25]. Briefly, larvae were allowed to feed on food supplemented with plenty of yeast (fed) or floated in 20% sucrose solution for 3 hours (starved). Fat bodies were dissected, stained with Lysotracker Red (Invitrogen) and DAPI in PBS for 5 minutes, and photographed live. A Zeiss Axioimager M2 microscope equipped with an Apotome2 unit and a Plan-NeoFluar 40× 0.75 NA objective was used to capture images with Axiovision software. Primary images were processed in Adobe Photoshop to produce final figures. Tissues were processed for transmission electron microscopy analysis as described previously [6,18,25]. Images were captured on a Jeol JEM-1011 microscope using an Olympus Morada 11 megapixel camera and iTEM software (Olympus).

### Statistics

Autophagic structures in ultrastructural images, Lysotracker Red (LTR) and mCherry-Atg8a puncta were manually counted in Photoshop. Statistical evaluation of GFP-CL1, p62 and anti-Atg8a images was carried out using ImageJ (NIH) as described [21,25]. As proteasome RNAi and *sima* overexpressing cells were usually smaller than control cells, the numbers of dots per GFP-positive clone area or control tissue area were counted. To avoid errors owing to cell size differences, dot numbers shown in figures are normalized to control cells, that is, if the average size of GFP-positive cells was 10% of control cells and contained 5 dots on average, then the normalized number of dots per cell is 50 [21].

Images taken from multiple individual larvae per genotype (n) as indicated in figure legends were evaluated. Data were analyzed in PASW Statistics 18 (IBM). After testing for normality of data distribution, p values were calculated by Student’s t tests or Mann-Whitney u tests in pairwise comparisons for normal or non-normal distribution data, respectively. We used ANOVA for multiple comparisons of normal distribution data and Kruskal-Wallis test for non-normal distribution data. Bar charts show average values and standard deviations. Regression analyses were also done in SPSS.

LDH-GFP fluorescence was detemined with ImageJ (NIH) software. Individual larvae were selected in the greyscale GFP channel, and the mean gray value was measured. Backgrund fluorescence from near each larva was subtracted from the mean gray value.

## Competing interests

The authors declare that they have no competing interests.

## Authors’ contributions

PL, AV, MS and GJ designed experiments. PL, AV, KP, PN, ZS, GJ carried out experiments. PL, AV, KP, ZS, GJ analyzed data. AV prepared figures. PL, GJ wrote the manuscript. All authors read and approved the final manuscript.

## Supplementary Material

Additional file 1: Figure S1 Aggregates of GFP-CL1 accumulate in proteasome RNAi cells. A-D) Expression of transgenic RNAi constructs in mosaic animals for *Prosα1* (A), *Prosα5* (B), *Prosβ2* (C), and *Rpt1* (D) results in accumulation of GFP-CL1 aggregates in larval fat body cells. Scale bar in A equals 20 μm for A-D. Click here for file

Additional file 2: Figure S2 Aggregates of p62 and ubiquitinated proteins accumulate in proteasome RNAi cells. A-H) Knockdown of *Prosα1* (A, B), *Prosα5* (C, D), *Prosβ2* (E, F), and *Rpt1* (G, H) leads to the formation of large aggregates containing ubiquitinated proteins (A, C, E, G) and p62 (B, D, F, H). Boxed areas in A-H are shown enlarged. Scale bar in A equals 20 μm for A-H. Click here for file

Additional file 3: Figure S3 Proteasome RNAi upregulates *p62* transcription. Systemic depletion of *Prosβ2* or *Rpt1* leads to increased transcription of *p62* relative to controls in RT-PCR experiments. Click here for file

Additional file 4: Figure S4 Cell size decreases upon proteasome inactivation. Regression analysis reveals that GFP-CL1 level inversely changes with cell size in proteasome RNAi cells. Spearman’s correlation coefficient = -0.728, p < 0.001, R^2^ Linear = 0.332. Click here for file

Additional file 5: Figure S5 Proteasome RNAi enhances starvation-induced autophagy. A-D) Knockdown of *Prosα1* (A), *Prosα5* (B), *Prosβ2* (C), and *Rpt1* (D) leads to increased punctate LTR staining in fat body cell clones of starved larvae compared to control non-GFP cells. Boxed areas in A-D are shown enlarged. Scale bar in A equals 20 μm for A-D. Click here for file

Additional file 6: Figure S Lamp1-GFP partially colocalizes with LTR. Colocalization of the reporter Lamp1-GFP that labels primary lysosomes, late endosomes and digesting lysosomes is not complete with LTR, a dye that stains acidic structures only. Boxed area is shown enlarged. Scale bar equals 20 μm. Click here for file

Additional file 7: Figure S7 Proteasome RNAi induces autophagy in well-fed cells. A-H) Knockdown of *Prosα1* (A, B), *Prosα5* (C, D), *Prosβ2* (E, F), and *Rpt1* (G, H) induces the formation of LTR-positive autolysosomes in fat body cell clones (marked by Lamp1-GFP expression) compared to surrounding non-GFP control cells in well fed larvae (A, C, E, G), and also leads to increased generation of Atg8a-positive autophagosomes (B, D, F, H) in fat body cell clones of well fed larvae. Boxed areas in A-H are shown enlarged. Scale bar in A equals 20 μm for A-H. Click here for file

Additional file 8: Figure S8 Punctate LTR staining is increased by proteasome inactivation. Regression analysis reveals that punctate LTR staining inversely changes with cell size in proteasome RNAi cells. R^2^ = 0.101, P < 0.001. The linearized equation of the curve is the following: ln(y) = ln(a) + b*ln(x), where a = -0.012 ± 0.002; p < 0.001 and b = 1.015 ± 0.002. Click here for file

Additional file 9: Figure S9 Overexpressed Atg8a reporters are captured into p62 aggregates in proteasome RNAi cells. A-D) Overexpressed GFP-Atg8a is incorporated into large p62-positive aggregates in *Prosα1* (A), *Prosα5* (B), *Prosβ2* (C), and *Rpt1* (D) RNAi cells. Boxed areas in A-D are shown enlarged. Scale bar in A equals 20 μm for A-D. Click here for file

Additional file 10: Figure S10 Autophagic flux is enhanced upon genetic inactivation of the proteasome. A-H) Knockdown of *Prosα1* (A, B), *Prosα5* (C, D), *Prosβ2* (E, F), and *Rpt1* (G, H) cells expressing the tandemly tagged mCherry-GFP-Atg8a reporter induces the formation of mCherry-labeled autolysosomes in fat body cell clones in well fed larvae (A, C, E, G). The lysosome inhibitor chloroquine blocks autophagy-dependent quenching of GFP, as now most puncta are positive for both mCherry and GFP (B, D, F, H). Boxed areas in A-H are shown enlarged. Scale bar in A equals 20 μm for A-H. Click here for file

Additional file 11: Figure S11 Protein aggregates and autophagic structures form in fat body cells undergoing *Rpn2* RNAi. Depletion of *Rpn2* in whole fat bodies (mediated by the cg-Gal4 driver) results in the formation of protein aggregates (agg), double-membrane autophagosomes (arrow) and digesting autolysosomes (arrowhead) in cells. Scale bars equal 1 μm. Click here for file

Additional file 12: Figure S12 Accumulation of p62 aggregates is increased by proteasome inactivation. Regression analysis reveals that accumulation of p62 aggregates inversely changes with cell size in proteasome RNAi cells. Spearman’s correlation coefficient = -0.806, P < 0.001, R^2^ Linear = 0.412. Click here for file

Additional file 13: Figure S13 LTR-positive autolysosomes do not colocalize with p62 and Atg8a reporters. A, B) p62-GFP (A) and GFP-Atg8a (B) display practically no colocalization with the lysosome marker LTR. Boxed areas in A and B are shown enlarged. Scale bar in A equals 20 μm for A and B. Click here for file

Additional file 14: Figure S14 Caspases are not activated in proteasome or *Vhl* RNAi or sima overexpressing cells. A-H) No active caspase 3 immunoreactivity is detected in *Prosα1* (A), *Prosα5* (B), *Prosβ2* (C), *Rpt1* (D) and *Rpn2* (E) RNAi fat body cells. (F) Similarly, no active caspase 3 immunolabeling is detected in *Rpt1* RNAi cells (marked by Lamp1-GFP) in brains. Note that several control cells positive for active caspase 3 (arrowheads) are seen in this panel. Similarly, overexpression of sima (G) or depletion of *Vhl* (H) does not lead to activation of caspase 3 in fat body cells either. Boxed areas in A-H are shown enlarged. Scale bar in A equals 20 μm for A-H. Click here for file

Additional file 15: Figure S15 The hypoxia reporter LDH-GFP is activated in proteasome or *Vhl* RNAi animals. Systemic depletion of *Prosβ2*, *Rpn2*, *Rpt1* or *Vhl* all lead to a similar upregulation of the transcriptional hypoxia reporter transgene *LDH-GFP*, compared to control larvae. Per cent values refer to GFP fluorescence. Click here for file

Additional file 16: Figure S16 List of Drosophila stocks used in this study. Click here for file

Additional file 17: Figure S17 Original images for gels and western blots. Black boxes highlight cropped regions, which are shown in image panels as indicated. Click here for file
