# Supplementary figures and images for: Impaired proteasomal degradation enhances autophagy via hypoxia signaling in Drosophila
Source: BMC Cell Biol. 2013 Jun 25;14:29. doi: 10.1186/1471-2121-14-29 (PMC3700814; doi:10.1186/1471-2121-14-29)

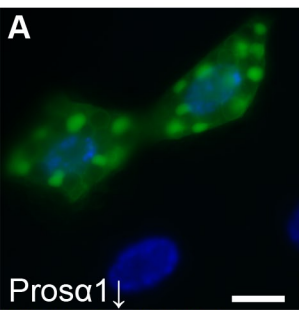

DNA, GFP-CL1

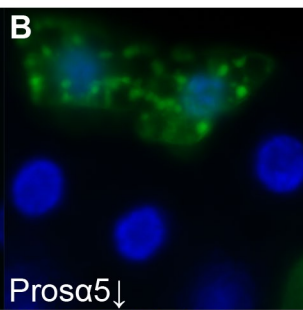

Prosα5↓  
DNA, GFP-CL1

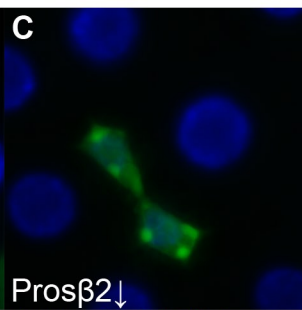

Prosβ2↓  
DNA, GFP-CL1

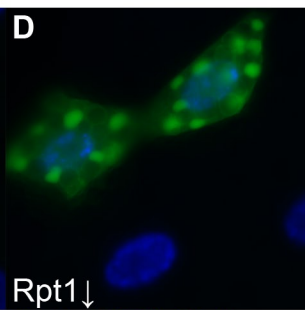

Rpt1↓  
DNA, GFP-CL1

Supplement: Additional file 1: Figure S1 — Aggregates of GFP-CL1 accumulate in proteasome RNAi cells. A-D) Expression of transgenic RNAi constructs in mosaic animals for Prosα1 (A), Prosα5 (B), Prosβ2 (C), and Rpt1 (D) results in accumulation of GFP-CL1 aggregates in larval fat body cells. Scale bar in A equals 20 μm for A-D. [file 1471-2121-14-29-S1.pdf]

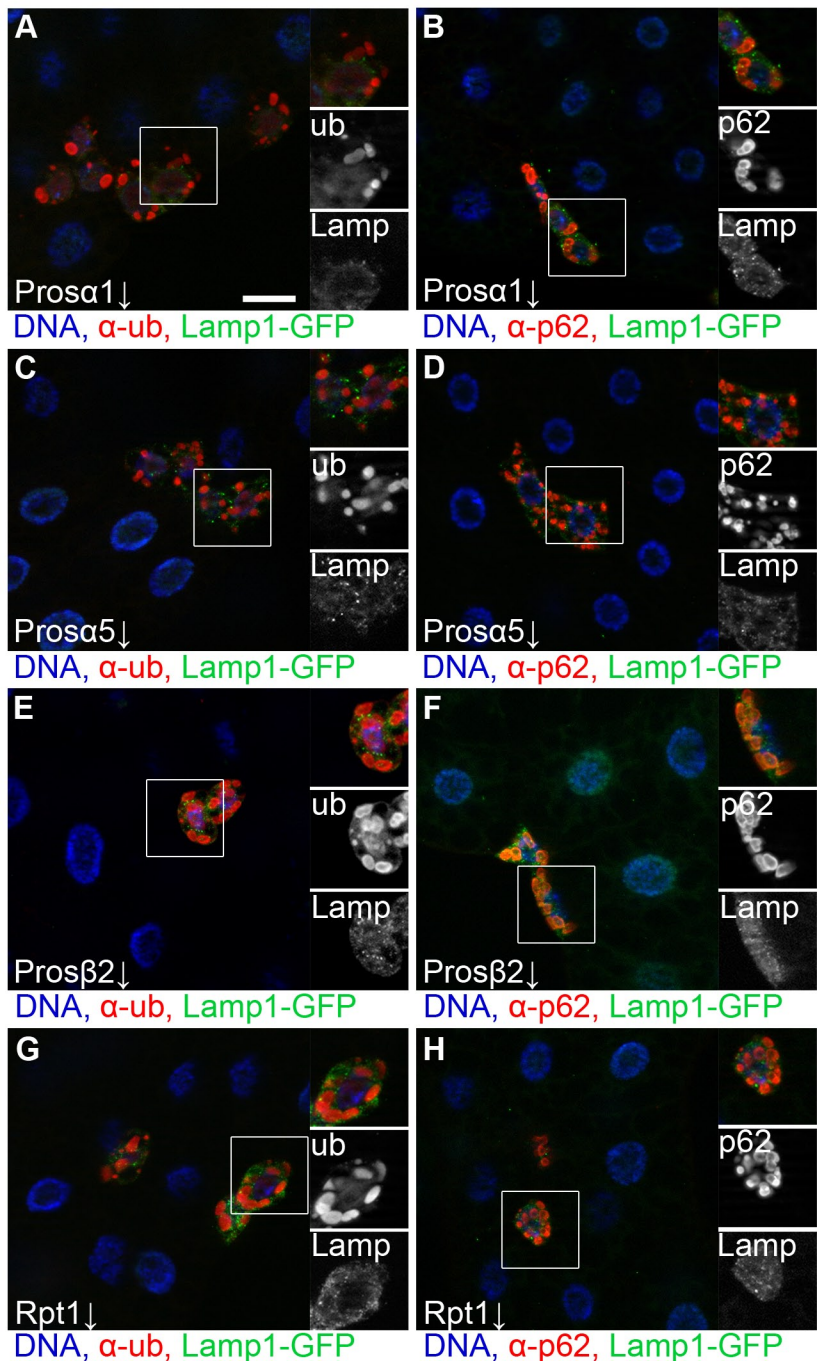

Supplement: Additional file 2: Figure S2 — Aggregates of p62 and ubiquitinated proteins accumulate in proteasome RNAi cells. A-H) Knockdown of Prosα1 (A, B), Prosα5 (C, D), Prosβ2 (E, F), and Rpt1 (G, H) leads to the formation of large aggregates containing ubiquitinated proteins (A, C, E, G) and p62 (B, D, F, H). Boxed areas in A-H are shown enlarged. Scale bar in A equals 20 μm for A-H. [file 1471-2121-14-29-S2.pdf]

control

Prosβ2↓

Rpt1↓

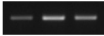

p62

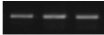

Actin5c

Supplement: Additional file 3: Figure S3 — Proteasome RNAi upregulates p62 transcription. Systemic depletion of Prosβ2 or Rpt1 leads to increased transcription of p62 relative to controls in RT-PCR experiments. [file 1471-2121-14-29-S3.pdf]

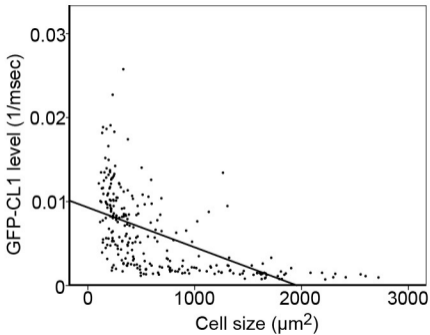

Supplement: Additional file 4: Figure S4 — Cell size decreases upon proteasome inactivation. Regression analysis reveals that GFP-CL1 level inversely changes with cell size in proteasome RNAi cells. Spearman’s correlation coefficient = -0.728, p < 0.001, R2 Linear = 0.332. [file 1471-2121-14-29-S4.pdf]

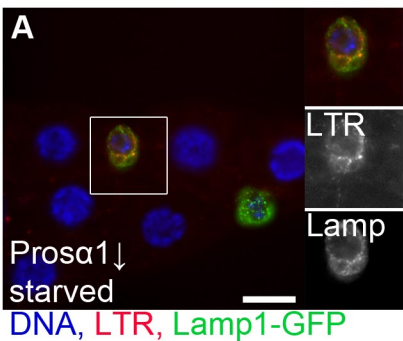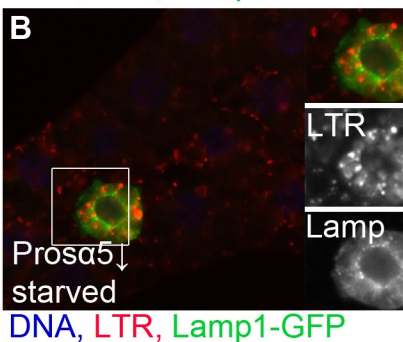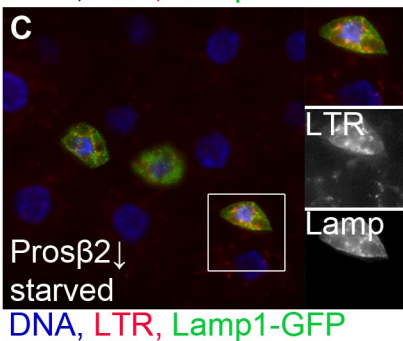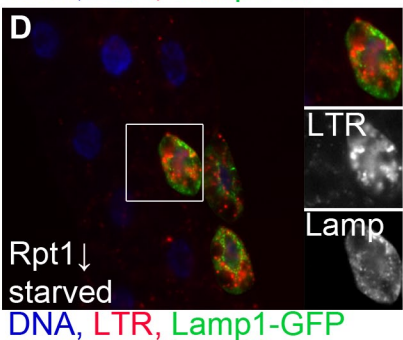

Supplement: Additional file 5: Figure S5 — Proteasome RNAi enhances starvation-induced autophagy. A-D) Knockdown of Prosα1 (A), Prosα5 (B), Prosβ2 (C), and Rpt1 (D) leads to increased punctate LTR staining in fat body cell clones of starved larvae compared to control non-GFP cells. Boxed areas in A-D are shown enlarged. Scale bar in A equals 20 μm for A-D. [file 1471-2121-14-29-S5.pdf]

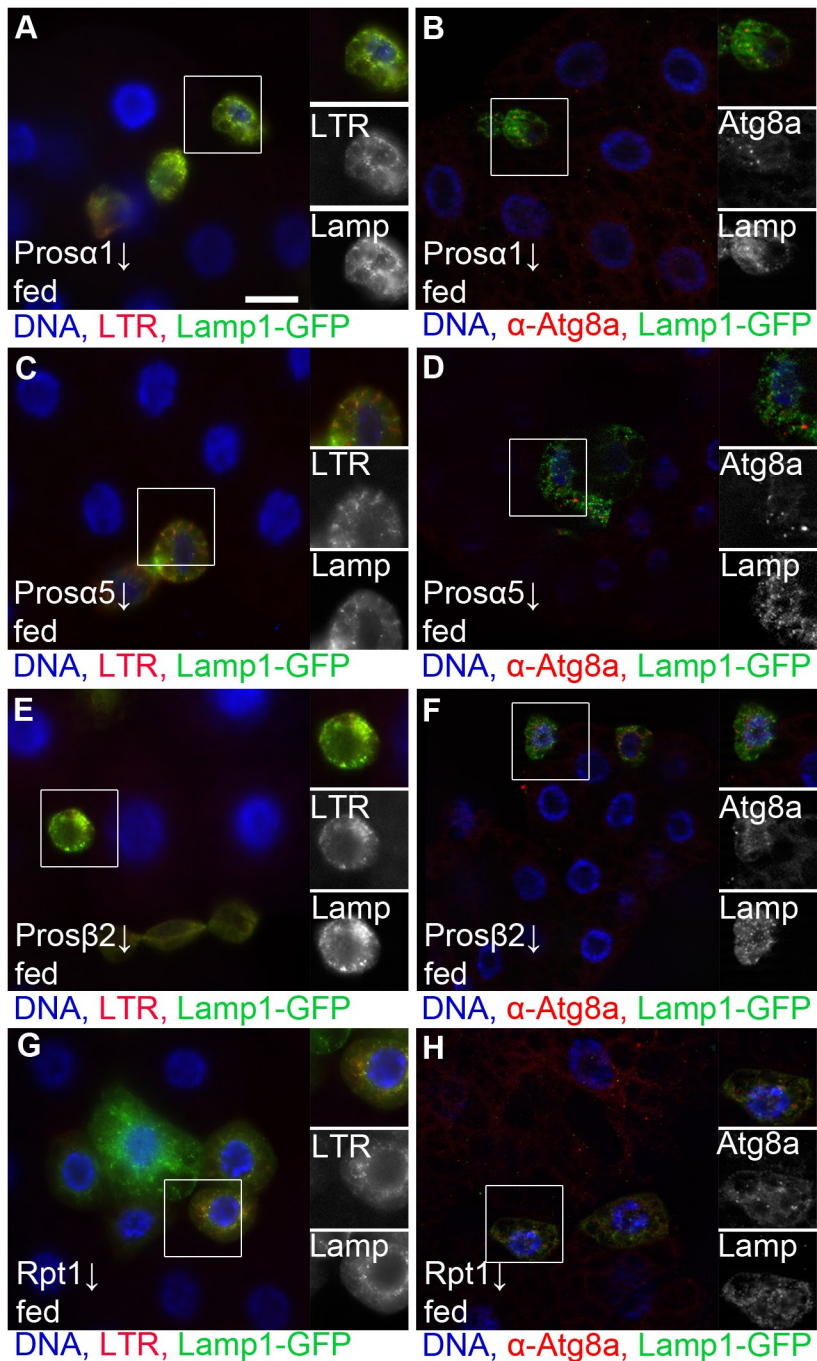

Supplement: Additional file 7: Figure S7 — Proteasome RNAi induces autophagy in well-fed cells. A-H) Knockdown of Prosα1 (A, B), Prosα5 (C, D), Prosβ2 (E, F), and Rpt1 (G, H) induces the formation of LTR-positive autolysosomes in fat body cell clones (marked by Lamp1-GFP expression) compared to surrounding non-GFP control cells in well fed larvae (A, C, E, G), and also leads to increased generation of Atg8a-positive autophagosomes (B, D, F, H) in fat body cell clones of well fed larvae. Boxed areas in A-H are shown enlarged. Scale bar in A equals 20 μm for A-H. [file 1471-2121-14-29-S7.pdf]

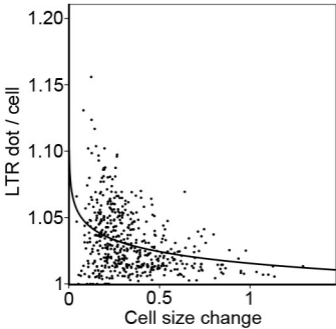

Supplement: Additional file 8: Figure S8 — Punctate LTR staining is increased by proteasome inactivation. Regression analysis reveals that punctate LTR staining inversely changes with cell size in proteasome RNAi cells. R2 = 0.101, P < 0.001. The linearized equation of the curve is the following: ln(y) = ln(a) + b*ln(x), where a = -0.012 ± 0.002; p < 0.001 and b = 1.015 ± 0.002. [file 1471-2121-14-29-S8.pdf]

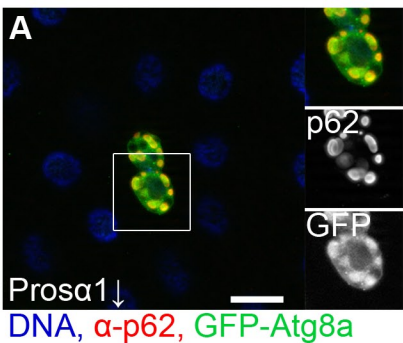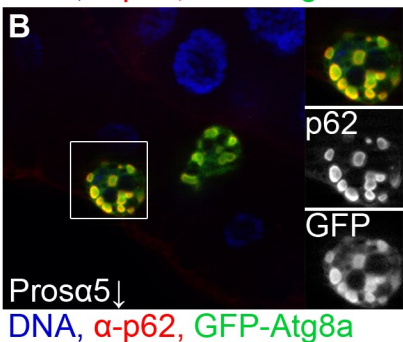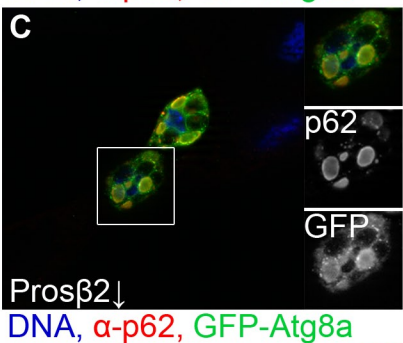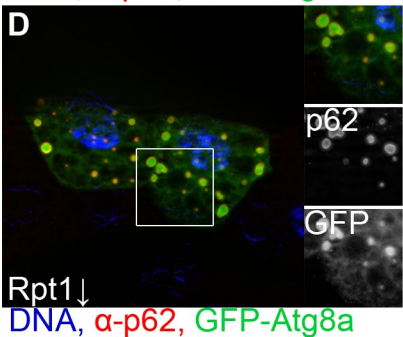

Supplement: Additional file 9: Figure S9 — Overexpressed Atg8a reporters are captured into p62 aggregates in proteasome RNAi cells. A-D) Overexpressed GFP-Atg8a is incorporated into large p62-positive aggregates in Prosα1 (A), Prosα5 (B), Prosβ2 (C), and Rpt1 (D) RNAi cells. Boxed areas in A-D are shown enlarged. Scale bar in A equals 20 μm for A-D. [file 1471-2121-14-29-S9.pdf]

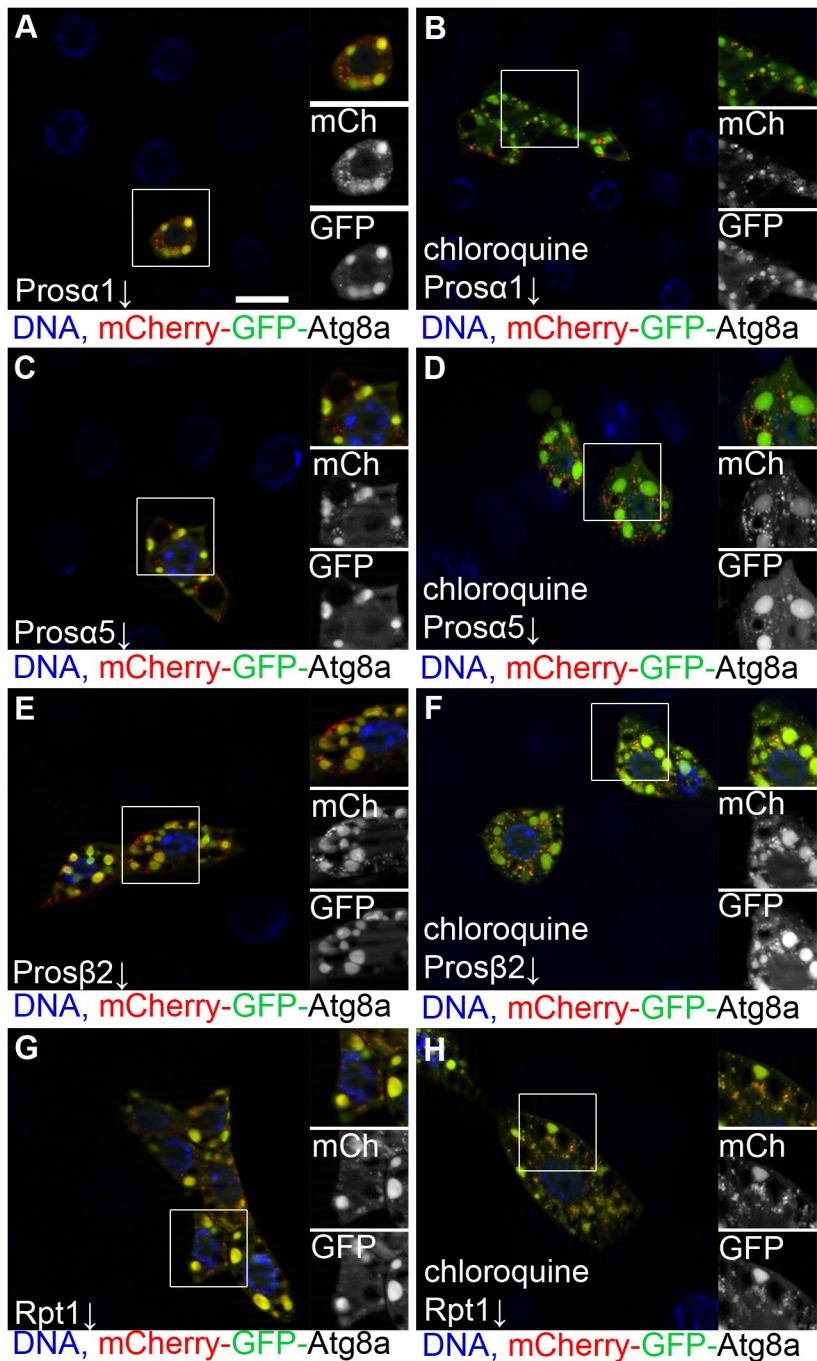

Supplement: Additional file 10: Figure S10 — Autophagic flux is enhanced upon genetic inactivation of the proteasome. A-H) Knockdown of Prosα1 (A, B), Prosα5 (C, D), Prosβ2 (E, F), and Rpt1 (G, H) cells expressing the tandemly tagged mCherry-GFP-Atg8a reporter induces the formation of mCherry-labeled autolysosomes in fat body cell clones in well fed larvae (A, C, E, G). The lysosome inhibitor chloroquine blocks autophagy-dependent quenching of GFP, as now most puncta are positive for both mCherry and GFP (B, D, F, H). Boxed areas in A-H are shown enlarged. Scale bar in A equals 20 μm for A-H. [file 1471-2121-14-29-S10.pdf]

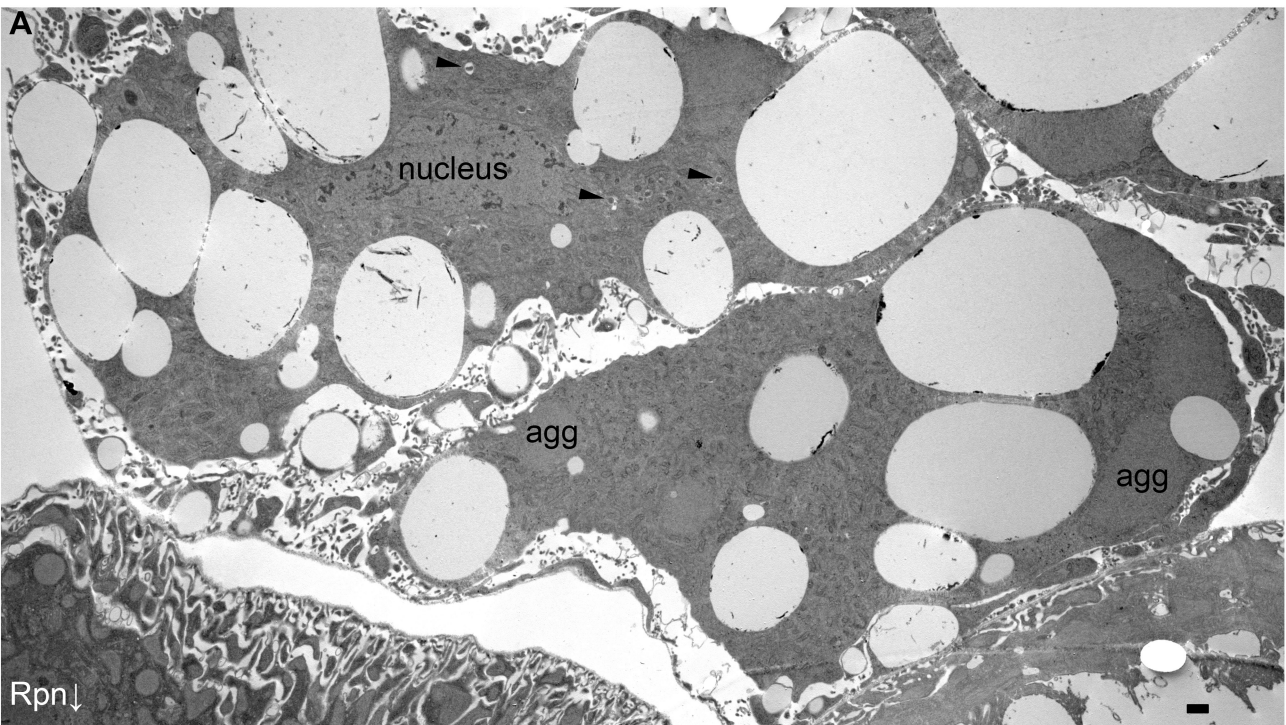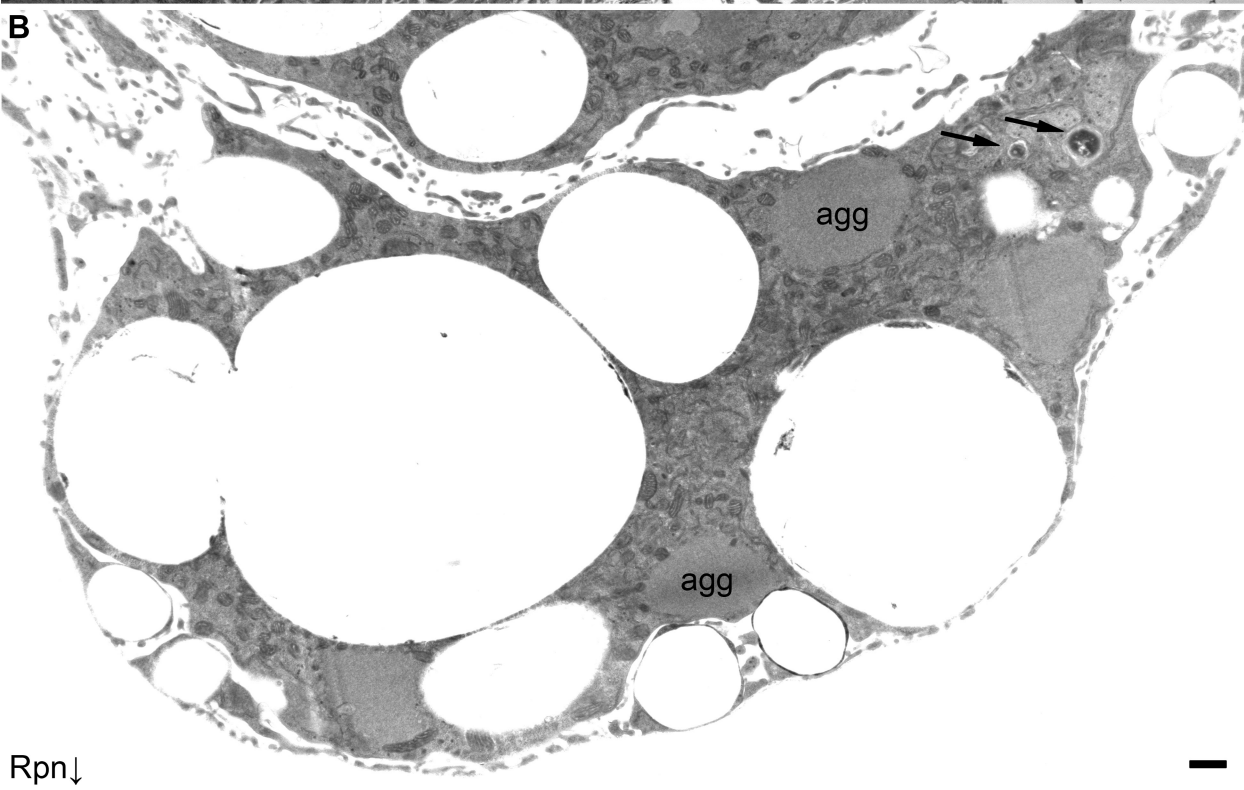

Supplement: Additional file 11: Figure S11 — Protein aggregates and autophagic structures form in fat body cells undergoing Rpn2 RNAi. Depletion of Rpn2 in whole fat bodies (mediated by the cg-Gal4 driver) results in the formation of protein aggregates (agg), double-membrane autophagosomes (arrow) and digesting autolysosomes (arrowhead) in cells. Scale bars equal 1 μm. [file 1471-2121-14-29-S11.pdf]

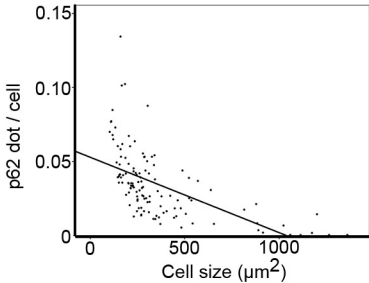

Supplement: Additional file 12: Figure S12 — Accumulation of p62 aggregates is increased by proteasome inactivation. Regression analysis reveals that accumulation of p62 aggregates inversely changes with cell size in proteasome RNAi cells. Spearman’s correlation coefficient = -0.806, P < 0.001, R2 Linear = 0.412. [file 1471-2121-14-29-S12.pdf]

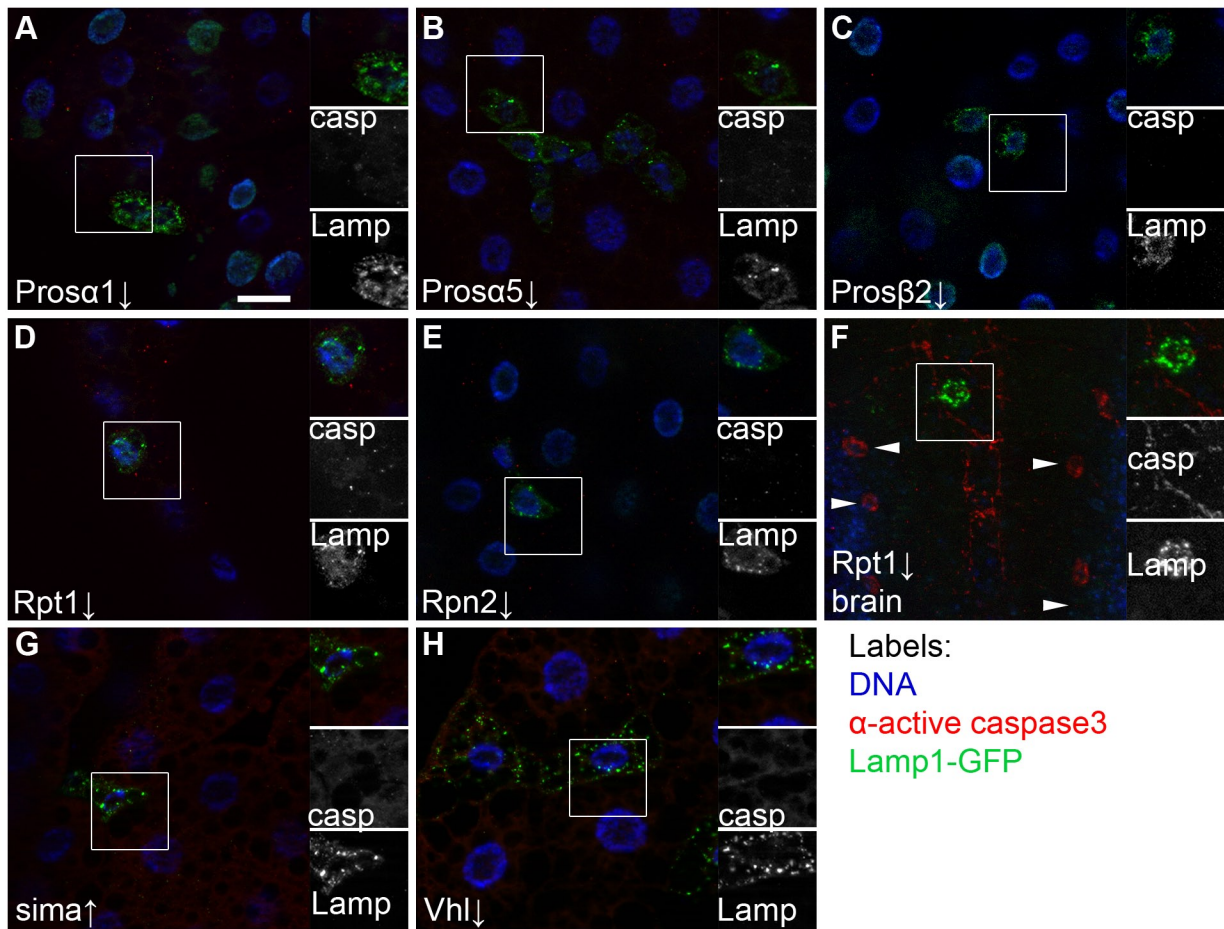

Supplement: Additional file 14: Figure S14 — Caspases are not activated in proteasome or Vhl RNAi or sima overexpressing cells. A-H) No active caspase 3 immunoreactivity is detected in Prosα1 (A), Prosα5 (B), Prosβ2 (C), Rpt1 (D) and Rpn2 (E) RNAi fat body cells. (F) Similarly, no active caspase 3 immunolabeling is detected in Rpt1 RNAi cells (marked by Lamp1-GFP) in brains. Note that several control cells positive for active caspase 3 (arrowheads) are seen in this panel. Similarly, overexpression of sima (G) or depletion of Vhl (H) does not lead to activation of caspase 3 in fat body cells either. Boxed areas in A-H are shown enlarged. Scale bar in A equals 20 μm for A-H. [file 1471-2121-14-29-S14.pdf]

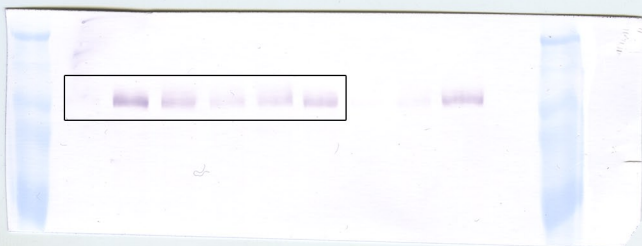

Figure 2F:  
p62

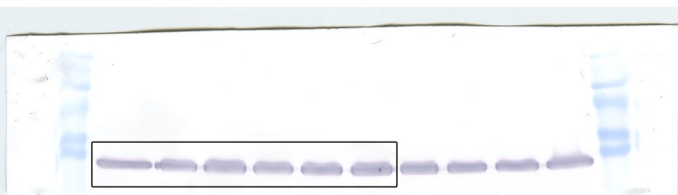

Figure 2F:  
Tubulin

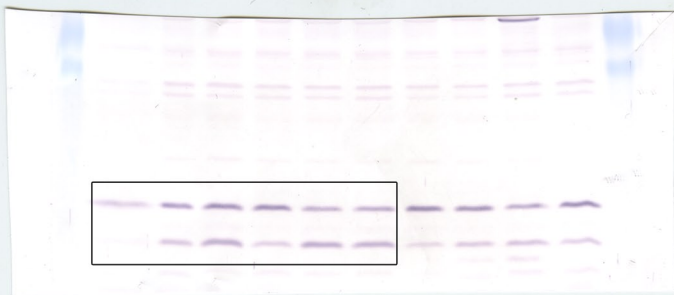

Figure 2F:  
Atg8a

Additional file 3: S3.pdf

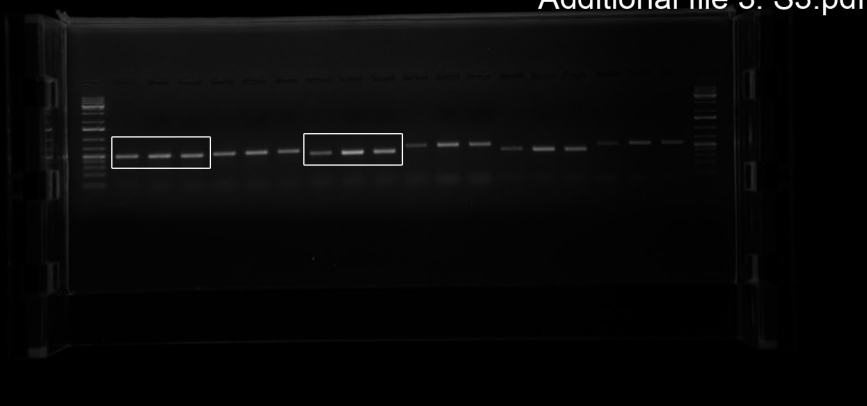

Figure 4L

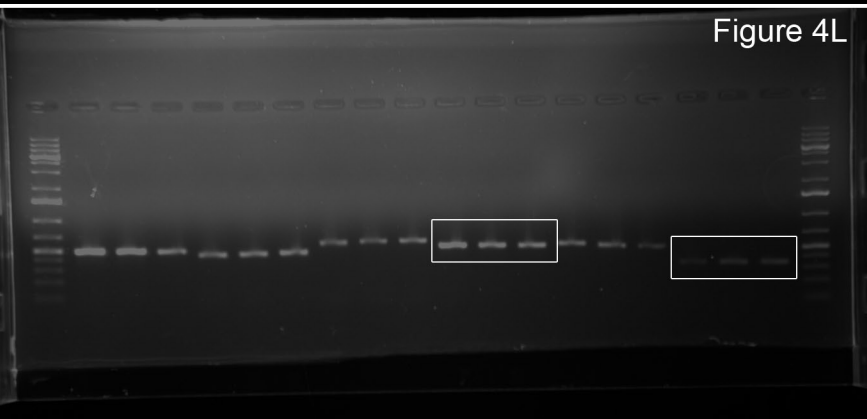

Supplement: Additional file 17: Figure S17 — Original images for gels and western blots. Black boxes highlight cropped regions, which are shown in image panels as indicated. [file 1471-2121-14-29-S17.pdf]
