# Supplementary material for: Impaired proteasomal degradation enhances autophagy via hypoxia signaling in Drosophila
Source: BMC Cell Biol. 2013 Jun 25;14:29. doi: 10.1186/1471-2121-14-29 (PMC3700814; doi:10.1186/1471-2121-14-29)
Supplement: Additional file 6: Figure S — Lamp1-GFP partially colocalizes with LTR. Colocalization of the reporter Lamp1-GFP that labels primary lysosomes, late endosomes and digesting lysosomes is not complete with LTR, a dye that stains acidic structures only. Boxed area is shown enlarged. Scale bar equals 20 μm. [file 1471-2121-14-29-S6.pdf]

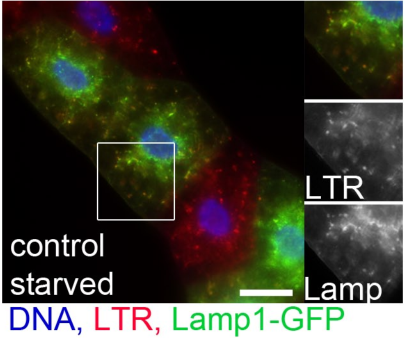

Genotype	Colocalizing dots / Lamp-1 dots counted	Colocalization %
control	143/200	76
Prosα1↓	176/200	88
Prosα5↓	132/200	66
Prosβ2↓	92/200	46
Rpt1↓	106/200	53
Rpn2↓	162/200	81
