# Supplementary material for: Impaired proteasomal degradation enhances autophagy via hypoxia signaling in Drosophila
Source: BMC Cell Biol. 2013 Jun 25;14:29. doi: 10.1186/1471-2121-14-29 (PMC3700814; doi:10.1186/1471-2121-14-29)
Supplement: Additional file 13: Figure S13 — LTR-positive autolysosomes do not colocalize with p62 and Atg8a reporters. A, B) p62-GFP (A) and GFP-Atg8a (B) display practically no colocalization with the lysosome marker LTR. Boxed areas in A and B are shown enlarged. Scale bar in A equals 20 μm for A and B. [file 1471-2121-14-29-S13.pdf]

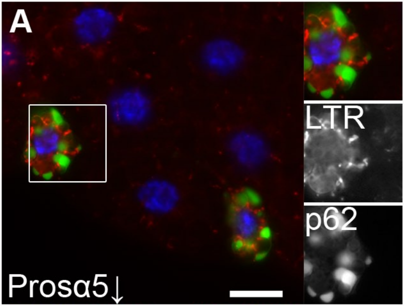

DNA, LTR, p62-GFP

Genotype	Colocalizing dots / LTR dots counted	Colocalization %
control	14/200	7
Prosα1↓	8/200	4
Prosα5↓	6/200	3
Prosβ2↓	3/200	1.5
Rpt1↓	3/200	1.5
Rpn2↓	13/200	6.5

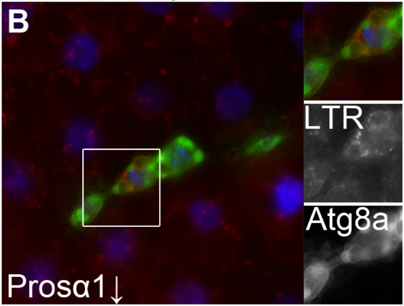

DNA, LTR, GFP-Atg8a

Genotype	Colocalizing dots / LTR dots counted	Colocalization %
control	170/200	85
Prosα1↓	4/200	2
Prosα5↓	3/200	1.5
Rpt1↓	11/200	5.5
Rpn2↓	6/200	3
