# Supplementary material for: Impaired proteasomal degradation enhances autophagy via hypoxia signaling in Drosophila
Source: BMC Cell Biol. 2013 Jun 25;14:29. doi: 10.1186/1471-2121-14-29 (PMC3700814; doi:10.1186/1471-2121-14-29)
Supplement: Additional file 15: Figure S15 — The hypoxia reporter LDH-GFP is activated in proteasome or Vhl RNAi animals. Systemic depletion of Prosβ2, Rpn2, Rpt1 or Vhl all lead to a similar upregulation of the transcriptional hypoxia reporter transgene LDH-GFP, compared to control larvae. Per cent values refer to GFP fluorescence. [file 1471-2121-14-29-S15.pdf]

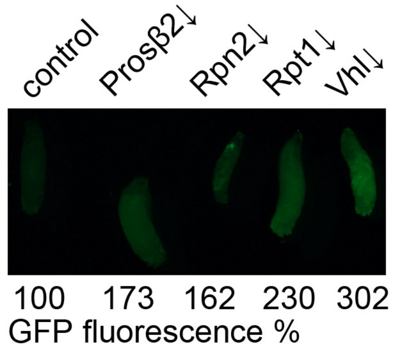
control
Prosβ2↓
Rpn2↓
Rpt1↓
Vhl↓
100
173
162
230
302
GFP fluorescence %
