# Supplementary material for: Impaired proteasomal degradation enhances autophagy via hypoxia signaling in Drosophila
Source: BMC Cell Biol. 2013 Jun 25;14:29. doi: 10.1186/1471-2121-14-29 (PMC3700814; doi:10.1186/1471-2121-14-29)
Supplement: Additional file 16: Figure S16 — List of Drosophila stocks used in this study. [file 1471-2121-14-29-S16.pdf]

**stock name**	**CG number**	**source**	**genotype/transformant ID**
control		BDSC	w[1118]
Prosα1↓	CG18495	VDRC	GD49681
Prosα3T↓	CG1736	VDRC	GD32889
Prosα5↓	CG10938	VDRC	KK108380
Prosα6↓	CG30382	BDSC	JF02711
Prosα7↓	CG1519	VDRC	KK102016
Pros25↓	CG5266	VDRC	KK101409
Pros28.1↓	CG3422	VDRC	KK105712
Pros29↓	CG9327	VDRC	KK104373
Prosβ2↓	CG3329	VDRC	KK103575
Prosβ3↓	CG11981	VDRC	KK100561
Prosβ4↓	CG17331	VDRC	GD19079
Prosβ5↓	CG12323	VDRC	KK107628
Prosβ7↓	CG12000	VDRC	KK101990
Pros26↓	CG4097	VDRC	KK105673
Pros45↓	CG1489	VDRC	KK100620
Rpt1↓	CG1341	VDRC	KK108834
Rpt3↓	CG16916	VDRC	KK100681
Rpt4↓	CG3455	BDSC	HMS00661
Tbp-1↓	CG10370	BDSC	HMS00417
Mov34↓	CG3416	VDRC	KK108573
Rpn1↓	CG7762	VDRC	KK103939
Rpn2↓	CG11888	VDRC	KK106457
Rpn5↓	CG1100	VDRC	GD18676
Rpn7↓	CG5378	VDRC	KK101467
Rpn9↓	CG10230	VDRC	KK103733
Rpn11↓	CG18174	BDSC	HMS00071
Pomp↓	CG9324	VDRC	KK100628
sima↑	CG7951	BDSC	UAS-sima
Vhl↓	CG13221	VDRC	KK108920
sima↓1	CG7951	BDSC	JF02105
sima↓2	CG7951	BDSC	HMS00832
sima↓3	CG7951	BDSC	HMS00833
p62↓	CG10360	BDSC	HMS00551
p62↓↓	CG10360	VDRC, BDSC	KK108193, HMS00938
Atg1DN↑	CG10967	Scott 2007	UAS-Atg1[KQ]
Atg1↓	CG10967	BDSC	JF02273
Vps34DN↑	CG5373	Juhasz 2008	UAS-Vps34[KD]
Atg9↓	CG3615	BDSC	JF02891
Atg4DN↑	CG4428	Pircs 2012	UAS-Atg4[DN]
Atg12↓	CG10861	BDSC	JF02704
Atg18b↓	CG8678	VDRC	KK100536
BNIP3↓	CG5059	VDRC	KK107493
mCD8-GFP		BDSC	hs-Flp, Act>CD2>Gal4, mCD8-GFP
GFP-CL1		Udai Pandey	UAS-GFP-CL1
Lamp1-GFP		our lab	hs-Flp; UAS-Lamp1GFP; Act>CD2>Gal4, UAS-Dcr2
mCherry-GFP-Atg8a		our lab	hs-Flp; UAS-mCherry-GFP-Atg8a; Act>CD2>Gal4, UAS-Dcr2
GFP-Atg8a		our lab	hs-Flp; UAS-GFP-Atg8a; Act>CD2>Gal4, UAS-Dcr2
mCherry-Atg8a, GFP clones		our lab	hs-Flp; UAS-Dcr2; Act>CD2>Gal4, UAS-GFPnls, r4-mCherry-Atg8a
LDH-GFP		Pablo Wappner	LDH-GFP
